# Knowledge, Perceptions, Attitudes, and Practices of Dog and Cat Owners Regarding Skin Tumors: A Cross-Sectional Study

**DOI:** 10.3390/vetsci12111020

**Published:** 2025-10-22

**Authors:** Cláudia Cardoso, Rita Files, Filipe Silva, Patricia Barbedo, Justina Prada, Isabel Pires

**Affiliations:** 1 Department of Veterinary Sciences, University of Trás-os-Montes and Alto Douro, 5000-801 Vila Real, Portugal; clauscardoso@utad.pt (C.C.); ritafiles2000@gmail.com (R.F.); fsilva@utad.pt (F.S.); alexandrabarbedo@gmail.com (P.B.); ipires@utad.pt (I.P.); 2 Associate Laboratory for Animal and Veterinary Sciences (AL4AnimalS), Animal and Veterinary Research Centre (CECAV), University of Trás-os-Montes and Alto Douro, 5000-801 Vila Real, Portugal

**Keywords:** skin tumor, dog, cat, risk factors, owner awareness, KAP, survey

## Abstract

**Simple Summary:**

Skin tumors are one of the most common types of cancer in dogs and cats, often influenced by factors such as sun exposure, genetics, and age. Because pets share the same environment as their owners, understanding what owners know and do about these tumors is essential for early detection and treatment. In this study, we surveyed 420 pet owners in Portugal to assess their knowledge, attitudes, and practices regarding pet ownership. Most owners were aware that pets can develop skin tumors, but many were unaware of the most common tumor types and associated risk factors. While most said they would take their pet to a veterinarian if they noticed suspicious skin changes, few regularly check their pets’ skin or use sun protection. Owners who had pets with previous tumors or a family history of skin cancer showed better knowledge and more proactive behaviors. These results reveal the need for better education and communication to help owners detect and prevent skin tumors early, thereby improving the health and wellbeing of dogs and cats.

**Abstract:**

Skin tumors are among the most common neoplasms in dogs and cats, sharing biological and environmental risk factors with human cancers. Owners play a critical role in early detection, yet little is known about their knowledge and attitudes. This study aimed to assess the knowledge, perceptions, attitudes, and practices of Portuguese pet owners regarding skin tumors in companion animals. An online cross-sectional survey was completed by 420 respondents. Overall, awareness of risk factors such as sun exposure and age was relatively high, but most owners were unable to identify specific tumor types or locations. Only one-quarter believed skin tumors are curable, while the majority expressed uncertainty. Women, those with multiple or long-term pet ownership, and individuals with family or personal experience of cancer showed greater knowledge and more proactive behaviors. However, a gap between knowledge and practice still remains. These findings underscore the need for targeted educational strategies to enhance owners’ health literacy, facilitate early detection, and promote timely veterinary care.

## 1. Introduction

Cancer in companion animals, particularly dogs and cats, has become an increasing global concern due to the rising number of diagnosed cases. This condition is among the leading causes of morbidity and mortality in these species, driving research aimed at understanding its incidence, risk factors, and clinical management. Epidemiological research conducted in various parts of the world has yielded valuable insights into tumors in these animals, with variations depending on the sample size, geographical region, and inclusion criteria employed [1,2,3,4,5,6].

Beyond their impact on animal health, spontaneous tumors in dogs and cats exhibit remarkable histopathological and biological similarities to human cancers, making them valuable models in comparative oncology. These similarities contribute to the development of new therapeutic approaches and a deeper understanding of tumor biology in both species. Tumor types of particular relevance to human oncology include osteosarcomas, mammary carcinomas, oral melanomas, oral squamous cell carcinomas, soft tissue sarcomas, and malignant non-Hodgkin’s lymphoma [1,4,7,8,9]. While biological parallels exist, environmental and lifestyle factors also play a critical role in the occurrence of cancer in both humans and pets. Because dogs and cats share the same living environment as their owners, they are exposed to many of the same carcinogenic agents. Identifying these risk factors is essential for prevention and timely treatment. Known contributors include exposure to environmental pollutants, ultraviolet radiation, pesticides, and even passive smoking resulting from human habits [10,11].

Among the neoplasms affecting dogs and cats, skin tumors are among the most common, representing a significant proportion of oncological diagnoses [6,7,12,13]. In dogs, approximately 20% to 30% of primary skin and subcutaneous tumors are malignant, whereas in cats, this proportion is even higher, ranging from 50% to 65% [14,15,16,17]. The most frequent skin tumors in dogs include lipomas, mast cell tumors, sebaceous gland tumors, and papillomas. In contrast, in cats, basal cell carcinomas, squamous cell carcinomas, and fibrosarcomas are most prevalent [8,13,16,18].

Owners play a central role in identifying dermatological abnormalities in their pets, as they are often the first to observe changes in the skin. Their ability to recognize risk factors and early clinical signs directly influences the time to diagnosis and initiation of therapy. However, it remains unclear whether most pet owners are aware of common risk factors, including sun exposure, genetic predisposition, and coat color, or whether they can identify clinical signs that may indicate a skin tumor [19].

Furthermore, understanding owners’ perceptions and attitudes toward cancer in pets is essential, as emotional responses, such as fear, fatalism, or misconceptions about treatment costs and prognosis, often influence decision-making. For instance, owners who believe that cancer is invariably fatal may delay seeking veterinary care or choose euthanasia over treatment. Conversely, those with higher awareness are more likely to adopt preventive measures, perform regular skin examinations, and promptly consult a veterinarian [20,21]. Given this context, identifying knowledge gaps, misconceptions, and behavioral patterns among pet owners is critical to guiding targeted educational strategies. Therefore, this study aimed to assess the knowledge, attitudes, perceptions, and practices of dog and cat owners in Portugal regarding skin tumors.

## 2. Materials and Methods

### 2.1. Study Design and Participants

A cross-sectional descriptive study was conducted using an anonymous online questionnaire created with Google Forms. The survey was designed to assess the knowledge, attitudes, and practices (KAP) of pet owners in Portugal, regarding skin tumors in dogs and cats.

The target population consisted of individuals aged 18 years or older residing in Portugal who owned at least one dog or cat. Participation was voluntary, and responses were collected between 26 July 2024, and 30 January 2025. The survey link was shared via email, social media platforms, and professional networks, using a QR code and a short description to encourage participation. Only individuals residing in Portugal and who owned at least one dog or cat were eligible to participate.

The minimum sample size was calculated using the formula proposed by Thrusfield (2018) [22], assuming a 95% confidence level (Z = 1.96), an expected prevalence of 50% (*p* = 0.5), and a margin of error of 5% (d = 0.05). Although the exact number of dog and cat owners in Portugal is not known, as one person may own more than one animal, a conservative estimate of 4.7 million was used, based on the total number of pet animals living in households (2.8 million dogs and 1.9 million cats, according to national data [23]). Although this figure likely overestimates the number of owners, it was chosen deliberately to avoid underestimation and ensure robust sample representation. Thus, a minimum of 384 valid responses was considered statistically representative.

Before dissemination, a pilot test was conducted with a small group of pet owners to evaluate the clarity, structure, and relevance of the questions. Feedback from the pilot phase was used to improve the final version of the questionnaire.

### 2.2. Instrument Structure

The questionnaire was divided into five main sections: 1: Informed Consent; 2: Sociodemographic Information—This section collected data on age, gender, nationality, region of residence (North, Center, Lisbon Metropolitan Area, Algarve, Alentejo, Autonomous Regions) and zip code, type of living environment (urban, rural, semi-urban), educational level, family status (whether they had children), and personal or family history of skin cancer; 3: Pet Ownership Profile—This section included questions about the number, type, and duration of pet ownership, as well as any previous experience with pets diagnosed with skin tumors; 4: Knowledge, Attitudes, Perceptions, and Practices—This section assessed participants’ understanding, beliefs, emotional perceptions, and practices related to skin tumors in companion animals.

### 2.3. Questionnaire Domains

The survey was structured based on the traditional KAP model (Knowledge, Attitudes, Practices), with an additional fourth domain—Perceptions—introduced to distinguish between factual understanding, evaluative beliefs, subjective interpretations, and real-world behaviors.

#### 2.3.1. Knowledge

This domain assessed participants’ factual knowledge regarding various aspects of skin tumors in dogs and cats. The questions were designed to evaluate awareness of the occurrence of skin cancer in companion animals, breed predisposition, the most common types of skin tumors in dogs and cats, their typical anatomical locations, and the causes and risk factors associated with these tumors. The identification of these causes and risk factors was based on a comprehensive review of the scientific literature (e.g., sun exposure, genetic predisposition, advanced age, virus). However, additional factors that are not consistently supported by evidence were also included to assess both knowledge about scientifically validated risks and potential misinformation that may influence owners’ decisions.

Knowledge regarding clinical signs and diagnostic tests was also explored. Additionally, participants were asked to indicate their main sources of information, including veterinarians, internet resources, friends/family, and printed media.

#### 2.3.2. Perceptions

This domain addressed participants’ subjective beliefs and emotional interpretations concerning skin tumors in companion animals. It encompassed questions regarding the perceived curability and preventability of skin tumors, their potential severity when left untreated, and the possibility of spontaneous resolution without medical intervention.

In addition, participants’ perceptions of risk were assessed, specifically their beliefs regarding the likelihood of their own pets developing skin tumors.

The domain also examined aspects of self-efficacy and responsibility, including the participants’ self-reported ability to identify suspicious skin lesions, the importance attributed to regular monitoring of their pets’ skin, and the intention to seek veterinary consultation in the event of detecting abnormalities.

Responses were obtained through closed-ended items, employing a three-point Likert scale (“Agree,” “Disagree,” “I don’t know”) for belief-related statements, and dichotomous options (“Yes” or “No”) for questions concerning self-assessed ability and perceived risk.

#### 2.3.3. Attitudes

This domain evaluated participants’ predisposition to act in response to situations related to skin tumors. Questions included willingness to seek veterinary care upon noticing suspicious skin changes and readiness to pay for treatment if their pet was diagnosed with a skin tumor.

#### 2.3.4. Practices

This final domain examined actual behaviors and routines related to the prevention, detection, and management of skin tumors in pets. It included questions on the frequency of skin checks and veterinary visits, exposure to sunlight, and the use of protective measures, as well as hygiene and grooming practices.

### 2.4. Data Analysis

Data were analyzed using IBM SPSS Statistics (Version 23). Descriptive statistics (frequencies and percentages) were used to summarize categorical variables.

Associations between sociodemographic characteristics, pet ownership profiles, and participants’ responses across the Knowledge, Perceptions, Attitudes, and Practices domains were assessed using the Chi-square (χ^2^) test. When more than 20% of expected cell counts were below five, or when both variables were dichotomous (2 × 2 tables), Fisher’s exact test was applied instead.

The normality of continuous variables (e.g., age) was evaluated using the Shapiro–Wilk test, which indicated a non-normal distribution (*p* < 0.05). Therefore, age was dichotomized based on the median (39 years).

A significance level of *p* < 0.05 was adopted for all analyses.

## 3. Results

A total of 420 valid responses were collected. As outlined in Section 2, the survey was structured according to the Knowledge, Attitudes, Perceptions, and Practices framework. Results are presented below by domain, preceded by a sociodemographic and contextual characterization of the sample.

### 3.1. Participant Profile

#### 3.1.1. Sociodemographic Data

Of the 420 individuals who completed the questionnaire, 397 (94.5%) identified themselves as Portuguese. Six participants were English, and five were either Spanish or Brazilian. The remaining respondents represented other nationalities, with only one individual per nationality.

**Figure 1:**
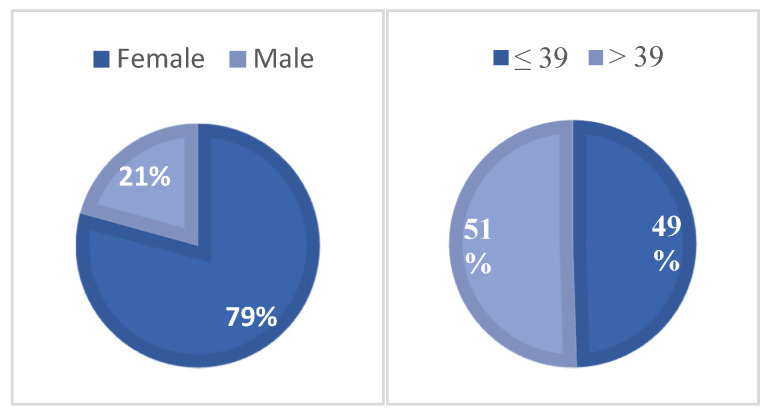
Distribution of respondents by sex (**left**) and by age group based on the median age of 39 years (**right**).

The majority of participants were female (*n* = 333; 79.3%), while 87 (20.7%) were male (Figure 1).

Participants ranged in age from 18 to 80 years, with a mean age of 39.14 years (SD = 15.0) and a median age of 39 years. For statistical purposes, age was categorized into two groups based on the median age (Figure 1).

**Figure 2:**
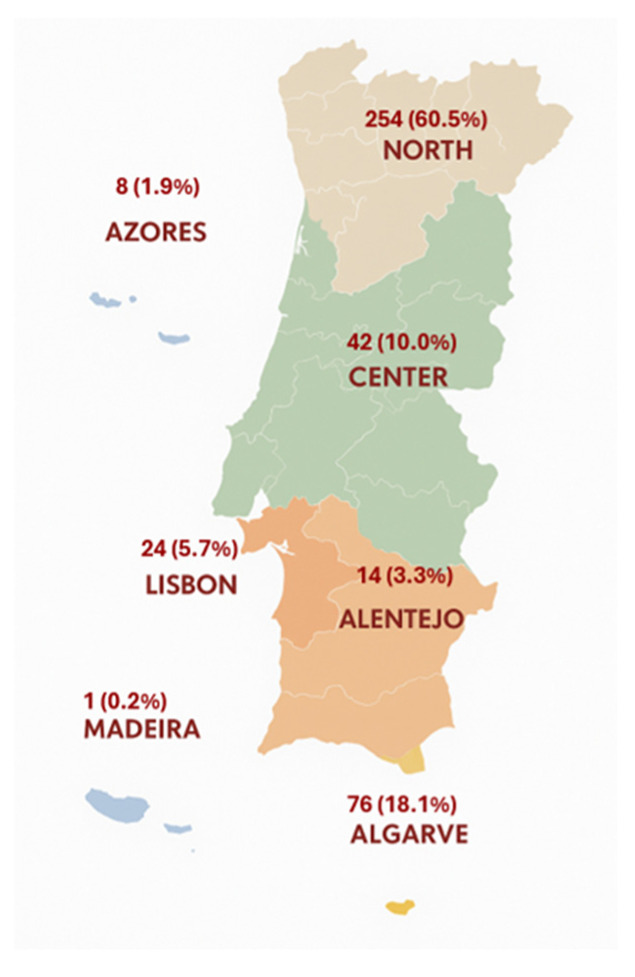
Geographical distribution of the owners who answered the questionnaire.

Regarding geographical distribution, most respondents resided in the Northern region, accounting for 60.5% (*n* = 254). This was followed by the Algarve, with 18.1% (*n* = 76), and the Central region, with 10.0% (*n* = 42). The Lisbon Metropolitan Area represented 5.7% (*n* = 24) of the participants, while the Alentejo region accounted for 3.3% (*n* = 14). The Autonomous Regions were less represented: 1.9% (*n* = 8) from the Azores and 0.2% (*n* = 1) from Madeira (Figure 2).

Concerning living environment, 211 participants (50.2%) reported living in urban areas, 105 (25.0%) in rural areas, and 104 (24.8%) in semi-urban areas.

Most participants held a bachelor’s degree (42.1%; *n* = 177), followed by secondary education (29.5%; *n* = 124), a master’s degree (19.5%; *n* = 82), and a doctorate (6.4%; *n* = 27). A small number reported having 5 to 9 years of schooling (1.9%; *n* = 8) or basic education (4th degree) (0.5%; *n* = 2).

When asked about having children, 28.6% (*n* = 120) reported having children, whereas 71.4% (*n* = 300) stated that they did not.

A family history of skin cancer was reported by 10.2% (*n* = 43) of participants, with melanoma being the most frequently mentioned. The remaining 89.8% (*n* = 377) indicated no known cases in their family.

#### 3.1.2. Pet Ownership Profile

A total of 61.9% owned more than one pet, while 38.1% had only one. Regarding species, 40.2% owned only dogs, 23.1% only cats, and 36.7% multiple types of animals. The most frequent combinations were dogs and cats (23.1%), followed by a combination of dogs, cats, and exotic animals.

Most participants (71.9%) had owned pets for over nine years, 23.1% for three to eight years, and 3.8% for less than two years (Table 1).

Of the 420 guardians interviewed, 32 (7.6%) reported that their pets had skin tumors, of which 25 (78.1%) were dogs and 7 (21.9%) were cats. The most frequently identified neoplasms were mast cell tumor (3 cases, 9.4%), sarcoma (3 cases, 9.4%), and squamous cell carcinoma (3 cases, 9.4%), followed by hemangioma (3 cases, 9.4%), carcinoma (2 cases, 6.3%), melanoma (1 case, 3.1%), adenoma (1 case, 3.1%), fibrosarcoma (1 case, 3.1%), histiocytoma (1 case, 3.1%), and undifferentiated tumors (2 cases, 6.3%). In the remaining cases, the tumor type was not identified by the guardians. Regarding dog breed, mixed-breed dogs (7 cases, 21.9%) were the most frequently affected, followed by French Bulldogs (4 cases, 12.5%), Labrador Retrievers (4 cases, 12.5%), Breton Spaniel (2 cases, 6.3%), and Portuguese Podengo (2 cases, 6.3%). Other isolated cases were observed in the Dalmatian, Miniature Schnauzer, Poodle, Rafeiro do Alentejo, and Shih Tzu breeds. All animals were European Shorthair cats (7 cases, 21.9%). The predominant age group was between 7 and 13 years, corresponding to 71.9% of the cases. Females were slightly more affected (18 cases, 56.3%) compared to males (14 cases, 43.7%). Concerning coat color, light-colored animals were predominant (27 cases, 84.4%), while dark-coated animals accounted for only 5 cases (15.6%).

### 3.2. Owners’ Knowledge About Skin Tumors in Dogs and Cats

#### 3.2.1. Knowledge About the Occurrence and Type of Skin Cancer in Animals

A considerable number of participants were either unaware (17.9%; *n* = 75) or uncertain (12.6%; *n* = 53) that skin cancer can affect animals. Women, owners with multiple pets, and those with longer pet ownership demonstrated greater awareness of this condition (*p* = 0.042, *p* = 0.001, and *p* = 0.004, respectively).

Most respondents (77.9%; *n* = 327) correctly recognized that some dog breeds are more predisposed to developing skin tumors, and 74.0% (*n* = 311) identified similar susceptibility in specific cat breeds. Awareness of canine breed predisposition was higher among younger owners (*p* < 0.001) and those with more than one animal (*p* = 0.046). For cats, significant associations were found with age (*p* < 0.001), education level (*p* = 0.013), number of pets (*p* = 0.002), and duration of ownership (*p* = 0.027).

When asked about specific tumor types, the most frequently recognized tumor types in dogs were mast cell tumors (15.7%; *n* = 66) and melanomas (8.3%; *n* = 35), whereas in cats, squamous cell carcinoma (14.3%; *n* = 60) and melanoma (3.6%; *n* = 15) were most often identified. Other tumor types mentioned, in descending order of frequency, included carcinomas, lymphoma, fibrosarcoma, soft tissue sarcoma, lipoma, histiocytoma, and hemangiosarcoma. However, most participants selected “I don’t know”—66.2% (*n* = 278) for dogs and 69.5% (*n* = 292) for cats. Uncertainty (“I don’t know”) about tumor type in dogs and cats was significantly more common among older (*p* < 0.001), male (*p* = 0.006), urban or semi-urban participants (*p* = 0.020; *p* = 0.016), single-pet owners (*p* = 0.053; *p* = 0.003), and those with shorter ownership duration (*p* = 0.004; *p* = 0.006). Participants living in rural areas were more likely to identify basal cell carcinoma as the most frequent canine skin tumor (*p* = 0.003).

When asked directly whether squamous cell carcinoma (SCC) is a type of skin cancer, 59.5% (*n* = 250) answered affirmatively, while 39.3% (*n* = 165) selected “I don’t know.

**Figure 3:**
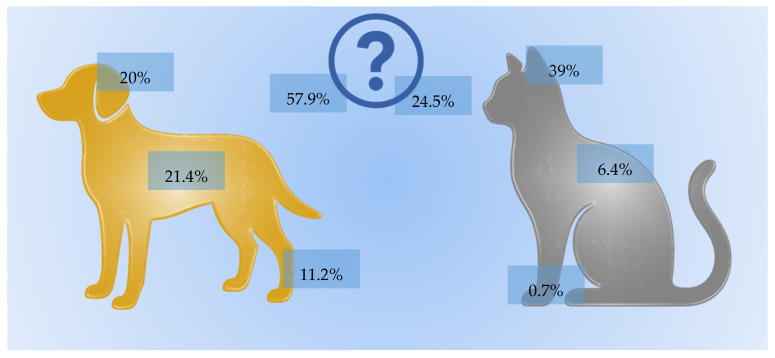
Distribution of participants’ responses regarding the most common anatomical sites for skin tumors in dogs and cats.

When asked about the most common anatomical sites of skin tumors in dogs, respondents most frequently selected the body/trunk (21.4%, *n* = 90), head (20.0%, *n* = 84), and limbs (11.2%, *n* = 47) (Figure 3). However, 57.9% (*n* = 243) answered “I don’t know,” particularly younger owners (*p* = 0.004) and those with a single pet (*p* = 0.011).

For cats, the head was most frequently cited as the common tumor site (39.0%, *n* = 164), followed by the body/trunk (6.4%, *n* = 27) and limbs (0.7%, *n* = 3). Over half of the respondents (54.5%, *n* = 229) selected “I don’t know” (Figure 3), more frequently among younger participants (*p* = 0.001), men (*p* = 0.002), and single-pet owners (*p* < 0.001).

When specifically asked about the ears of white cats, 49.8% (*n* = 209) of respondents acknowledged that tumors frequently occur in this area, while 49.5% (*n* = 208) were unsure. Women were more likely to identify high-risk locations, particularly the head (*p* = 0.001) and ears of white cats (*p* = 0.025). Likewise, younger participants (*p* = 0.004), single-pet owners (*p* < 0.001), and those with shorter ownership duration (*p* = 0.019) more often mentioned the head as a frequent tumor site. Recognition that white cats are commonly affected in the ears was also associated with having children (*p* = 0.011), a family history of cancer (*p* = 0.008), owning multiple animals (*p* < 0.001), and longer ownership duration (*p* = 0.036).

#### 3.2.2. Knowledge of Causes and Risk Factors Associated with Skin Tumors

Overall, awareness of well-established risk factors was relatively high. A large majority of respondents identified genetic predisposition (81.9%; *n* = 344), sun exposure (78.8%; *n* = 331), and old age (74.5%; *n* = 313) as significant contributors to the development of skin tumors. Light-colored fur was also widely recognized as a risk factor (68.1%; *n* = 286).

Perceptions of risk factors were influenced by participant profile. Younger owners were more likely to identify light-colored eyes (*p* = 0.045), personal or family history of cancer (*p* = 0.014), medical treatments (*p* < 0.001), lack of vaccination (*p* < 0.001), lack of deworming (*p* < 0.001), use of pipettes (*p* = 0.006), and use of disinfectants (*p* < 0.001) as risk factors. Women more frequently recognized sun exposure (*p* = 0.001) and light coat color (*p* < 0.001). Rural and semi-urban participants also cited sun exposure more often (*p* = 0.003). Having children was associated with broader risk perception, particularly regarding sun exposure (*p* = 0.019). A family history of cancer was associated with light-colored eyes (*p* = 0.018). Owners of multiple pets more often considered light coat color a risk factor (*p* = 0.008), while long-term owners more frequently mentioned disinfectant use (*p* = 0.011). Owners of pets previously affected by skin tumors were more likely to associate vaccination (*p* < 0.001) and disinfectant use (*p* = 0.017) with cancer risk.

The results revealed notable knowledge gaps regarding potential risk factors. Between 35–45% of respondents cited medical treatments, poor skin hygiene, and lack of external deworming as possible contributors, but were unsure of their actual role. High uncertainty was observed for the use of spot-on pipettes (57.9%, *n* = 243) and disinfectants (50.0%, *n* = 210). Similarly, many participants were uncertain about clipping (43.1%, *n* = 181) and frequent bathing (54.3%, *n* = 228). A summary is provided in Table 2.

A small proportion of respondents believed that male animals are more frequently affected (4.5%; *n* = 19), while the vast majority (79.5%; *n* = 334), indicated uncertainty about this potential correlation. Likewise, the association between guardians’ history of skin cancer and the risk of cancer in their animals was unclear to most participants, with 39.8% (*n* = 167) responding “don’t know”. When asked whether exposure to certain types of viruses could cause skin cancer in animals, the majority of participants stated they did not know (see Table 2).

#### 3.2.3. Knowledge About the Identification of Clinical Signs

**Figure 4:**
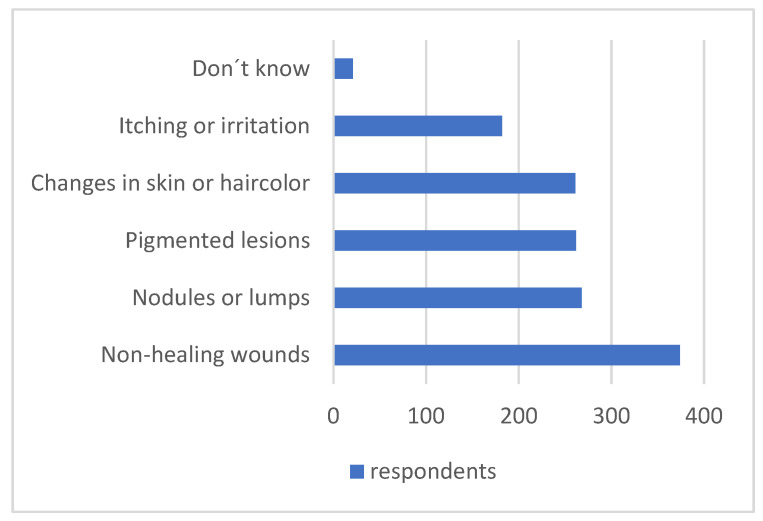
Frequency with which participants recognized possible signs associated with skin tumors in animals.

A considerable portion of respondents recognized suspicious clinical signs, and only 4.8% (*n* = 21) reported not knowing. The most frequently recognized signs were non-healing wounds (89.0%; *n* = 374), followed closely by nodules or lumps (63.8%; *n* = 268), pigmented lesions or moles (62.4%; *n* = 262), and changes in skin or hair color (62.1%; *n* = 261). Itching or irritation was acknowledged by 43.3% (*n* = 182) of participants (Figure 4).

Recognition of these clinical signs was significantly associated with participant characteristics. Younger owners were more likely to report changes in coat or skin color (*p* = 0.019), non-healing wounds (*p* = 0.021), nodules (*p* = 0.010), discoloration (*p* = 0.002), and itching or irritation (*p* = 0.005) as warning signs. However, they also selected “I don’t know” more frequently (*p* = 0.001). Recognition of itching or irritation was further associated with a family history of cancer (*p* = 0.039). Owners of pets with prior skin tumors were more likely to identify coat or skin color changes (*p* = 0.004), nodules (*p* = 0.023), discoloration (*p* = 0.020), and itching or irritation (*p* = 0.003). “I don’t know” responses were also more frequent among men (*p* = 0.038).

#### 3.2.4. Knowledge About Diagnosis

Regarding diagnostic methods, most participants (89.5%; *n* = 376) correctly identified biopsy as an essential tool for confirming diagnosis. In contrast, 16.2% (*n* = 68) believed that visual assessment alone could be used to diagnose skin cancer, while 49.0% (*n* = 206) disagreed, and 34.8% (*n* = 146) were uncertain. Misconceptions about visual diagnosis were more common among younger participants (*p* = 0.002), single-pet owners (*p* = 0.045), and those whose pets had previously developed skin tumors (*p* = 0.041).

#### 3.2.5. Main Information Sources

Veterinarians (62.1%; *n* = 283) and the internet (67.4%; *n* = 261) were the main sources of information, followed by books, magazines, and friends/family.

### 3.3. Perceptions

Participants expressed varied subjective beliefs and emotional interpretations regarding the diagnosis, treatment, and perceived risk of skin tumors in pets. Although 75.5% (*n* = 317) believed that skin tumors could be prevented, only 24.5% (*n* = 103) considered them generally curable, while 56.0% (*n* = 235) were uncertain. Belief in prevention was more frequent among owners with children (*p* = 0.022), long-term owners (*p* = 0.022), and those with a family history of skin cancer (*p* = 0.006) or pets previously diagnosed with tumors (*p* = 0.017). Owners of pets with prior tumors were also more likely to consider most tumors curable (*p* = 0.026).

Most respondents (81.2%, *n* = 341) agreed that untreated tumors could be fatal. Owners of pets previously diagnosed with tumors were more likely to share this belief (*p* = 0.034).

Most participants (78.3%, *n* = 329) disagreed with the statement that tumors can resolve without medical treatment, while only 1.2% (*n* = 5) believed they could, and 20.5% (*n* = 86) responded “don’t know.” Age was associated with this belief (*p* = 0.002), with younger owners more likely to disagree and older owners more often responding “don’t know.” Multiple-pet owners were also more likely to disagree that tumors can resolve without medical treatment (*p* = 0.019).

A majority of respondents (87.1%, *n* = 366) supported initiating treatment as soon as cancer is suspected to prevent disease progression, with only 1.9% (*n* = 8) disagreeing and 11.0% (*n* = 46) uncertain. Women (*p* = 0.040) and multiple-pet owners (*p* = 0.002) more strongly supported this necessity.

Most respondents (90.4%, *n* = 378) agreed that pets should be taken to a veterinarian if any skin abnormality is observed, whereas 5.3% (*n* = 22) disagreed and 4.3% (*n* = 18) were neutral. Owners with children were significantly more likely to value veterinary consultation (*p* < 0.001). Similarly, 88.8% (*n* = 371) agreed that regular monitoring of their pet’s skin is important, while 5.3% (*n* = 22) disagreed and 6.0% (*n* = 25) remained neutral. Owners with children also placed greater importance on routine skin checks (*p* < 0.001).

**Figure 5:**
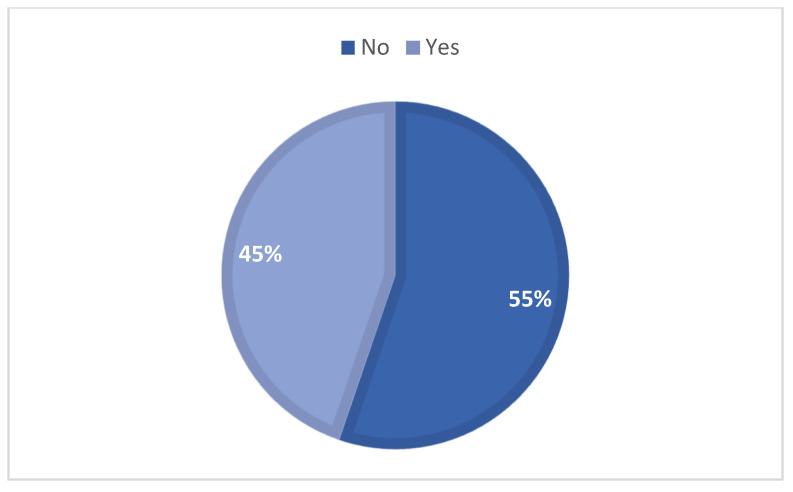
Owners’ responses on their ability to recognize clinical signs of skin tumors in animals, categorized as ‘Yes’ or ‘No’.

The majority of participants (55.0%, *n* = 232) reported not feeling capable of identifying suspicious skin lesions. However, 45.0% (*n* = 188) believed they could recognize such signs (Figure 5). The ability to identify suspicious lesions was associated with age (*p* < 0.001), number of pets (*p* = 0.002), and duration of ownership (*p* = 0.001), being more common among younger, multiple-pet, and long-term owners.

**Figure 6:**
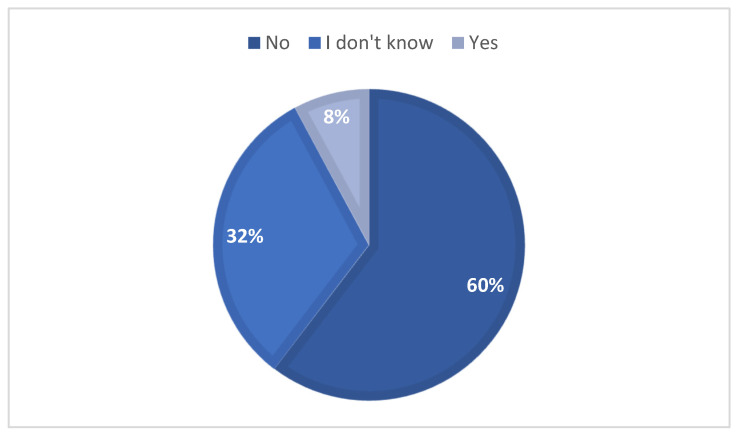
Owners’ perception of their animals being at risk of developing skin tumors, with responses categorized as ‘Yes’, ‘No’, or I don’t Know.

Most owners did not believe their pets were at risk of developing skin cancer (Figure 6). This perception of a high risk was associated with previous experience of pet tumors (*p* < 0.001) and was more common among long-term owners (*p* < 0.001).

### 3.4. Attitudes

Respondents demonstrated a generally serious and proactive attitude toward skin tumors in pets. Most (90.7%) stated they would take their pet to a veterinarian if skin changes were observed. Gender influenced these reactions (*p* = 0.028), with women more often indicating immediate veterinary consultation, while men were more likely to wait or seek information first. Previous experience with pet tumors was also significant (*p* = 0.005), as these owners reported more frequent and prompt veterinary visits.

When asked about willingness to pay for treatment, the majority expressed strong commitment: 44.8% (*n* = 188) stated they would do so without hesitation, and 49.5% (*n* = 208) would pay, though with financial limitations. A smaller proportion were unsure (4.5%; *n* = 19), and only 1.2% (*n* = 5) indicated they would not be willing to pay for treatment.

### 3.5. Practices

This domain analyzed the actual behaviors and routines of participants regarding the prevention, early detection, and management of skin tumors in companion animals.

#### 3.5.1. Skin Checks and Veterinary Visits

Regarding self-monitoring, 38.3% (*n* = 161) of owners reported frequently checking their pets’ skin, 40.7% (*n* = 171) did so occasionally, and 16.4% (*n* = 69) rarely performed checks. A minority (4.5%; *n* = 19) stated they never examined their pets. Owners whose pets had previously developed skin tumors were significantly more likely to report regular skin checks (*p* = 0.044).

When asked about veterinary visits, 57.1% (*n* = 240) of participants took their pets to the veterinarian twice or more per year, while 29.8% (*n* = 125) visited the veterinarian once annually. A smaller proportion sought care only when the animal was ill (7.4%; *n* = 31), or did not attend veterinary consultations every year (5.7%; *n* = 24). Routine veterinary care, including vaccination and deworming, was well established, with 78.6% (*n* = 330) of respondents reporting regular preventive visits, compared to 21.4% (*n* = 90) who only attended in case of illness.

#### 3.5.2. Sun Exposure and Protective Measures

Most animals were reported to spend time sunbathing during the day (75.7%; *n* = 318), although owners rarely specified the duration of exposure. Among these, 25.5% (*n* = 107) estimated exposure of up to 1 h, and 41.2% (*n* = 173) reported more than 1 h. A family history of cancer was associated with shorter reported sun exposure (*p* = 0.026).

Concerning sun protection, 61.0% (*n* = 256) stated they actively protected their pets from excessive sun, while 39.0% (*n* = 164) did not.

The use of sunscreen was minimal: only 4.5% (*n* = 19) applied it during peak sunlight hours, and 3.3% (*n* = 14) used it outdoors in the summer. The vast majority, 92.1% (*n* = 387), reported never using sunscreen on their pets.

#### 3.5.3. Hygiene and Grooming Practices

Regarding hygiene, 60.0% (*n* = 252) of owners reported bathing their pets, most commonly less than once per month (38.1%, *n* = 160). Only 6.0% (*n* = 25) bathed their pets more than once per month, and 19.3% (*n* = 81) did so monthly. A family history of cancer was associated with higher bathing frequency (*p* = 0.025).

A total of 76.2% (*n* = 320) of respondents brushed their pets, with 36.9% (*n* = 155) doing so more than once per month, while 23.8% (*n* = 100) never brushed them. Brushing frequency was associated with ownership duration (*p* = 0.031) and the presence of children in the household (*p* = 0.021).

## 4. Discussion

Cancer is a significant cause of death in companion animals, especially dogs and cats, posing a growing challenge for veterinarians. Their shorter lifespans contribute to higher annual neoplasia rates than in humans [1,24,25]. Among these, skin tumors are most frequent, largely due to continuous exposure to environmental carcinogens [26,27,28].

Most veterinary oncology studies focus on clinical aspects, but owner perspectives are increasingly recognized as essential [29,30,31,32]. Using an adapted KAP approach, this study explored Portuguese pet owners’ knowledge, perceptions, attitudes, and practices regarding skin tumors in dogs and cats.

Most respondents were concerned and willing to seek veterinary care. However, important knowledge gaps remain in early detection, risk-factor awareness, and perceptions of prognosis.

A great number of respondents were women, urban residents, and highly educated, similar to other studies, which may have increased general awareness. However, only 69.5% knew that animals can develop skin tumors, and most could not name specific types. Frequent skin tumors, such as mast cell tumors, melanomas, and squamous cell carcinomas [33,34,35] were mentioned occasionally, suggesting selective and limited disease knowledge.

A similar pattern was observed in the recognition of risk factors in our study. Participants widely acknowledged Sun exposure and age as major contributors, while more specific factors, such as the susceptibility of white cats’ ears, were often underestimated. Genetic and chemical risk factors also generated considerable uncertainty among respondents. In the literature, sun exposure and age were recognized risk factors [16,19], with most cases between 6 and 14 years [19,36,37], though papillomas and histiocytomas occur in younger dogs [7,38,39]. Sex-related patterns have also been reported, with females more commonly affected in dogs and males in cats [7,38]. Several dog breeds, including Boxers, Terriers, Bullmastiffs, Basset Hounds, Weimaraners, Kerry Blue Terriers, and Norwegian Elkhounds, present increased risk, whereas no specific breed predisposition has been described in cats [19,40,41]. Moreover, owners’ awareness of viral oncogenesis remains low, despite papillomaviruses being well-established causes of skin tumors in both animals [42,43,44] and humans [45,46,47], underscoring the owner education.

One of the most concerning findings was the belief in curability; only 24.5% believed that skin tumors are treatable, reflecting fatalistic views that may delay care and reduce treatment adherence. Encouragingly, 89.5% recognized the importance of biopsy, and 87.1% supported early treatment to prevent progression.

Only 7.6% of respondents reported having had a pet diagnosed with a skin tumor. While this may reflect real prevalence rates, it could also be the result of underdiagnosis, unawareness, or recall bias. The characteristics of the affected animals, light-colored coats, neutered status, and an age range of 7 to 13 align with the scientific literature [25,48]. Owners with prior experience of pet skin tumors demonstrated greater knowledge, stronger preventive attitudes, and more proactive behaviors, including increased awareness of risk factors, early detection, and regular veterinary checks. These findings suggest that direct experience plays a key role in improving health literacy and promoting behavioral change. Because pets are integral family members [49,50], direct experience with skin oncological disease, human or animal, appears to heighten risk perception and preventive behavior [51,52,53,54].

A family history of skin cancer increased attentiveness to UV and genetic risks, mirroring human studies where fear or loss enhances vigilance [55,56]. This bidirectional influence, where owners draw strength from pets and are more protective in turn, has important implications for veterinary education and public health messaging.

Other sociodemographic variables also influenced results. Women showed greater awareness and proactive behavior. Younger owners recognized environmental risks more, and those with multiple pets practiced more frequent checks and vet visits. These patterns suggest that species-specific and experience-driven strategies may be effective in tailoring educational efforts to meet the individual needs of each species.

Although veterinarians are trusted, only 62.1% cited them as their main information source, slightly below the internet (67.4%). This gap highlights the opportunity for veterinarians to strengthen education during routine visits, building on existing owner trust. Since most owners said they would visit a vet if suspicious signs appeared, this trust can be strengthened through better communication.

The study also revealed a noticeable gap between knowledge and practice. Although 75.7% of pets regularly sunbathe, only 7.9% of owners use sun protection. While 90.7% would seek veterinary care if skin changes were noticed, only 38.3% perform regular skin checks, and over half lack confidence in recognizing signs.

Owners’ emotional responses to pet cancer, shock, grief, and anxiety mirror those seen in human patients [57,58,59]. Pessimism about treatment persists, with only 24.5% believing in curability, yet this emotional bond offers an opportunity for communication through empathetic education [33,60,61,62].

Our findings emphasize the need for targeted, evidence-based education adapted to owners’ literacy levels and delivered through trusted channels such as veterinary clinics and community campaigns [63]. Addressing emotional and financial barriers, such as fear of diagnosis, cost concerns, and treatment misconceptions, is essential to encourage timely veterinary care [64,65,66]. From a public health perspective, incorporating behavioral insights into veterinary practice is crucial to bridging the gap between knowledge and action. Owner satisfaction and trust play a key role in treatment adherence and follow-up. Although our study did not directly assess satisfaction, the high proportion of respondents (90.7%) who would seek veterinary care after noticing a skin lesion suggests a foundation of trust that should be reinforced through consistent communication and empathy, as highlighted in previous qualitative studies [64]. However, persistent fears of anesthesia and surgery, particularly in older pets, may still delay care-seeking, as concerns about pain, recovery, or disfigurement often influence owners’ decisions [32].

Breed-specific risks also require greater attention. Purebred animals often show genetic predispositions to certain tumors [67,68,69,70], yet many owners remain unaware of these susceptibilities. Increasing awareness could promote more vigilant preventive practices and personalized veterinary monitoring.

Overall, this study reinforces the value of owner-centered surveys in identifying behavioral patterns, knowledge gaps, and emotional factors influencing animal care. Closing the gap between knowledge and action requires not only effective information delivery but also the integration of behavioral understanding into veterinary communication and education.

## 5. Conclusions

In conclusion, while Portuguese pet owners demonstrate generally responsible attitudes toward skin tumors in dogs and cats, significant knowledge gaps and shortcomings in preventive practices persist. Improving health literacy through continuous education, stronger communication between veterinary professionals and clients, and emotionally intelligent public health campaigns is essential. These efforts are key to promoting early detection, optimizing treatment outcomes, and ultimately enhancing the well-being of companion animals.

## Figures and Tables

**Table 1:** Frequency and Percentage of Responses from Guardians Regarding the Number of Pets and Duration of Pet Ownership.

Question/Response	*n*	%
How many pets do you have?	160	61.9%
One	260	38.1%
More than one		
What are the species of animals?		
Only Dog	169	40.2%
Only Cat	97	23.1%
Multiple types of animals	154	36.7%
Dogs and Cats	97	23.1%
Cats, Dogs, and Exotic animals	21	5%
Dogs, Cats, and Livestock	9	2.1%
Dogs and Exotic animals	9	2.1%
Cats and Exotic animals	8	1.9%
Dogs and Livestock	7	1.7%
Dogs, Cats, Exotic animals and Livestock	3	0.7%
How long have you had pets?		
Less than 2 years	16	3.8%
Between 3 and 8 years	97	23.1%
More than 9 years	302	71.9%
Not specified	5	1.2%

**Table 2:** Owners’ Responses to Statements Regarding the Risks and Causes of Skin Tumors in Dogs and Cats.

Etiological/Risk Factors	Yes *n* (%)	No *n* (%)	I Don’t Know *n* (%)
Sun exposure	331 (78.8%)	17 (4.0%)	72 (17.1%)
Light-colored fur	286 (68.1%)	21 (5.0%)	113 (26.9%)
Light colored eyes	174 (41.4%)	73 (17.4%)	173 (41.2%)
More frequent in males	19 (4.5%)	67 (16.0%)	334 (79.5%)
Owner with skin cancer	20 (4.8%)	233 (55.5%)	167 (39.8%)
Some medical treatments	171 (40.7%)	46 (11.0%)	203 (48.3%)
Lack of vaccination	132 (31.4%)	121 (28.8%)	167 (39.8%)
Lack of external deworming	150 (35.7%)	99 (23.6%)	171 (40.7%)
Use of spot-on pipettes	44 (10.5%)	133 (31.7%)	243 (57.9%)
Use of disinfectants	124 (29.5%)	85 (20.2%)	211 (50.2%)
Clipping	93 (22.1%)	145 (34.5%)	182 (43.3%)
Old age	313 (74.5%)	14 (3.3%)	93 (22.1%)
Genetic predisposition	344 (81.9%)	8(1.9%)	68 (16.2%)
Poor skin hygiene	190 (45.2%)	85 (20.2%)	145 (34.5%)
More than one bath per month	44 (10.5%)	147 (35.0%)	229 (54.5%)
Exposure to certain types of viruses	183 (43.6%)	18 (4.3%)	219 (52.1%)

## Data Availability

The original contributions presented in the study are included in the article/Supplementary Material; further inquiries can be directed to the corresponding author.

## Supplementary Materials

The following supporting information can be downloaded at: https://www.mdpi.com/article/10.3390/vetsci12111020/s1, File S1: Survey.
