# Supplementary material for: Knowledge, Perceptions, Attitudes, and Practices of Dog and Cat Owners Regarding Skin Tumors: A Cross-Sectional Study
Source: Vet Sci. 2025 Oct 22;12(11):1020. doi: 10.3390/vetsci12111020 (PMC12656767; doi:10.3390/vetsci12111020)
Supplement: Supplementary file 1 [file vetsci-12-01020-s001.zip › vetsci-3925883-supplementary.pdf]

Suplementary files:

Survey on Skin Tumors in Dogs and Cats:

This survey aims to assess the knowledge of pet owners of dogs and cats about skin tumors by gathering information on facts and perceptions about this topic among the Portuguese population. This survey is part of a doctoral thesis in Veterinary Sciences. It also aims to assess the impact of a skin tumor diagnosis on pet owners.

The results will be used only for academic/scientific purposes, and the survey is anonymous. Your participation in this project is confidential, and the research team is responsible for omitting any data that could potentially be identified. Your privacy and confidentiality of your personal data will be guaranteed, and refusal/withdrawal from the study will not result in any penalty.

1. **CONSENT**

   I give my consent for the use of the data obtained for scientific purposes. ☐

2. **OWNER INFORMATION**

   Age: ________________

   Gender

   - ☐ Male
   - ☐ Female
   - ☐ I prefer not to answer

   Nationality:

   - ☐ Portuguese
   - ☐ Other. Which one? _____________________

   Geographic region of residence:

   - ☐ North
   - ☐ Central
   - ☐ Lisbon Metropolitan Area
   - ☐ Alentejo
   - ☐ Algarve
   - ☐ Autonomous Region of Madeira
   - ☐ Autonomous Region of Azores

   Zip code of the region of residence: ______________________

   What the best describes the area where you live?

   - ☐ Rural
   - ☐ Urban
   - ☐ Semi-urban

   Level of education:

- ☐ Primary school (4th grade)
- ☐ Secondary school: 5th-9th grade
- ☐ Secondary school: 10th-12th grade
- ☐ Bachelor´s degree
- ☐ Master´s degree
- ☐ PhD

Do you have children?

- ☐ Yes
- ☐ No

~

Have you had or have a diagnosis of skin cancer in anyone in your family?

- ☐ Yes. Which cancer/tumor ? ______________________________
- ☐ No

3. PET OWNER PROFILE

How many pets do you have? _____________________

What species of animals do you have? ______________

How long have you had pets?

- ☐ < 2 years
- ☐ 3-8 years
- ☐ > 9 years

Has your pet ever been diagnosed with a skin tumor?

- ☐ No
- ☐ Yes. If yes please answer ___________________________

What species is your pet? ____________________________

What breed is your pet? ___________________________

What is your pet’s age? ___________________________

What is your pet’s sex? ___________________________

What is your pet’s predominant coat color? ___________________________

What type of tumor was diagnosed? ___________________________

4. KNOWLEDGE, ATTITUDES, PERCEPTIONS, AND PRACTICES

Are you aware of the existence of skin cancer in animals?

- ☐ Yes
- ☐ No
- ☐ I am not sure

What is the most common type of skin cancer in dogs? ________________

What is the most common type of skin cancer in cats? ________________

What is the most common location of skin cancer in dogs?

- ☐ Head (nose/ears)
- ☐ Trunk
- ☐ Limbs
- ☐ I don´t know

What is the most common location of skin cancer in cats?

- ☐ Head (nose/eares)
- ☐ Trunk
- ☐ Limbs
- ☐ I don´t know

Are the following risk factors or cause of skin cancer in dogs and/or cats? :

**Risk factor**	**Yes**	**No**	**I don´t know**
Sun exposure			
Have light fur			
Have clear eyes			
Being a male			
Owners with skin tumor			
Some medical treatments			
Lack of vaccination			
Lack of external deworming (against fleas and ticks)			
Deworming with spot on			
Use of disinfectants			
Clipping the pet			
Old age			
Genetic predisposition			
Poor skin hygiene			
More than 1 bath per month			
Exposure to certain types of viruses			

What signs do you consider suspicious of skin tumors in pets? (Select all that apply)

- ☐ Skin/Coat color change
- ☐ Wound that does not heal
- ☐ Nodules or lumps
- ☐ Itching or irritation
- ☐ Pigmented lesion/ mole
- ☐ I don´t know
- ☐ Other. Which one?________________________________

What is your opinion about the following sentences about skin cancer in dogs and cats (please answer without consulting the internet or other sources)?

Question	Yes	No	I don´t know
There are dog breeds that are more prone to developing skin tumors			
There are cat breeds that are more prone to developing skin tumors			
Squamous cell carcinoma is a type of skin tumor			
In white cats it is common in the ears			
A skin biopsy can help with the diagnosis			
Visual confirmation can confirm the diagnosis.			

Where did you get information about skin tumors in pets? (Select all that apply)

- ☐ From your veterinarian
- ☐ Internet
- ☐ Friends/ Family
- ☐ Books/ Magazines
- ☐ Other: Specify: ______________________

Regarding skin cancer in dogs and cats, what is your opinion? (Please answer without consulting the internet or other sources).

Question	Agree	Disagree	I don´t know
A skin tumor can be prevented			
Most skin tumors are curable			
A skin tumor can kill if left untreated			
Skin tumor usually heals without medical intervention			
If skin cancer is suspected, it must be treated to prevent the tumor from spreading to other locations			
It is important to monitor your dog's skin regularly.			
It is important to take your dog to the veterinarian if you notice an abnormality on the skin			

Do you feel able to identify signs of skin tumors in your pet?

- ☐ Yes
- ☐ No

Do you believe your pet is at high risk of developing skin tumors?

- ☐ Yes
- ☐ No
- ☐ I don´t know

How did you or would you react if you discovered or suspected a skin tumor in your pet?

- ☐ I would take him to the veterinarian immediately
- ☐ I would observe for a while before acting
- ☐ I would look for information online
- ☐ Other. Specify:____

Would you be willing to pay for expensive treatments for skin tumors in your pet?

- ☐ Yes, without hesitation
- ☐ Yes, but with limitations
- ☐ I don´t know
- ☐ No

Do you regularly check your pet's skin for abnormalities?

- ☐ Yes, often
- ☐ Occasionally
- ☐ Rarely
- ☐ Never

How many times a year do you take your pet to the veterinarian?

- ☐ Once
- ☐ Twice
- ☐ 3 or more times
- ☐ I don´t go every year
- ☐ Only if they get sick

What are the reasons for visits to the veterinarian? _________________

Does your pet spend time lying in the sun during the day?

- ☐ Yes. Approximately how many hours? A: __________________
- ☐ No

Do you protect your pet from excessive sun exposure?

- ☐ Yes
- ☐ No

Do you use sunscreen on your pet?

- ☐ Yes, whenever he goes outside in the summer
- ☐ Yes, during the hottest hours
- ☐ No, never
- ☐ Other: ____

Do you bathe your pet?

- ☐ Yes
- ☐ No

If yes, how often do you bathe it?

- ☐ Once a month
- ☐ Less than once a month
- ☐ More than once a month

Do you brush your pet?

- ☐ Yes
- ☐ No

If yes, how often do you brush it?

- ☐ Once a month
- ☐ Less than once a month
- ☐ More than once a month
